# Divergent responses to warming of two common co-occurring Mediterranean bryozoans

**DOI:** 10.1038/s41598-018-36094-9

**Published:** 2018-11-29

**Authors:** Marta Pagès-Escolà, Bernat Hereu, Joaquim Garrabou, Ignasi Montero-Serra, Andrea Gori, Daniel Gómez-Gras, Blanca Figuerola, Cristina Linares

**Affiliations:** 1 Department of Evolutionary Biology, Ecology and Environmental Sciences, Institut de Recerca de la Biodiversitat (IRBIO), University of Barcelona, Av. Diagonal 643, 08028 Barcelona, Spain; 2 Institute of Marine Sciences, ICM-CSIC, Pg. Marítim de la Barceloneta 37-49, 08003 Barcelona, Spain; 3 Smithsonian Tropical Research Institute, P.O. Box 0843-03092, Balboa, Republic of Panama

## Abstract

Climate change threatens the structure and function of marine ecosystems, highlighting the importance of understanding the response of species to changing environmental conditions. However, thermal tolerance determining the vulnerability to warming of many abundant marine species is still poorly understood. In this study, we quantified in the field the effects of a temperature anomaly recorded in the Mediterranean Sea during the summer of 2015 on populations of two common sympatric bryozoans, *Myriapora truncata* and *Pentapora fascialis*. Then, we experimentally assessed their thermal tolerances in aquaria as well as different sublethal responses to warming. Differences between species were found in survival patterns in natural populations, *P*. *fascialis* showing significantly lower survival rates than *M*. *truncata*. The thermotolerance experiments supported field observations: *P*. *fascialis* started to show signs of necrosis when the temperature was raised to 25–26 °C and completely died between 28–29 °C, coinciding with the temperature when we observed first signs of necrosis in *M*. *truncata*. The results from this study reflect different responses to warming between these two co-occurring species, highlighting the importance of combining multiple approaches to assess the vulnerability of benthic species in a changing climate world.

## Introduction

Marine ecosystems are highly affected by climate change, with impacts predicted to increase in the coming years^1–3^. Specifically, climatic projections of global sea surface temperature predict 0.3–4.8 °C increase by the end of the 21^st^ century, depending on the CO_2_ emissions scenario^4^. In addition to the expected steady increase in temperature, in recent years there has been an increase in the frequency of heat waves, causing mass mortality events in marine ecosystems and affecting a wide variety of species such as gorgonians, sponges, algae and fishes in temperate and tropical seas^2,5–7^. Increases in recurrence of these mortalities can lead to population declines and widespread shifts in species distributions, which are currently occurring in all ecosystems as a consequence of environmental changes^8,9^.

The fundamental niche of marine species is determined by their thermal tolerances, where their functional traits raise the optimal values, and an increase of temperature can affect negatively their physiological and demographic processes^10–12^. Due to climate change, in recent years there has been an increment of species that are frequently exposed to conditions over their thermotolerance limits, as the case of coral bleaching events^13,14^ or mass mortalities of Mediterranean populations of gorgonians^5,6^. As a result, species with low tolerance to warming are at the greatest risk of local extinction because of their limited thermoregulatory ability^15,16^. Related to this, the mortality of non-thermotolerant habitat-forming or key species can have serious consequences on the entire community, reducing species richness and structural complexity of prominent habitats such as kelp forests or coral reefs^1,17^.

The Mediterranean Sea is a biodiversity hotspot, highly threatened by climate change^18,19^. To understand the response of Mediterranean marine species to global warming, several studies have performed thermotolerance experiments in multiple co-occurring Mediterranean benthic species, such as anthozoans, revealing highly divergent levels of sensitivity^20–22^. In some cases, these divergences occur at population level, as in shallow populations of the red coral *Corallium rubrum* (Linnaeus, 1758), where some populations can be more tolerant to an increase of temperature^23^. However, while most of the thermotolerance preference studies have focused on charismatic taxa, such as gorgonians or corals, there is a lack of knowledge about other abundant benthic organisms.

**Figure 1:**
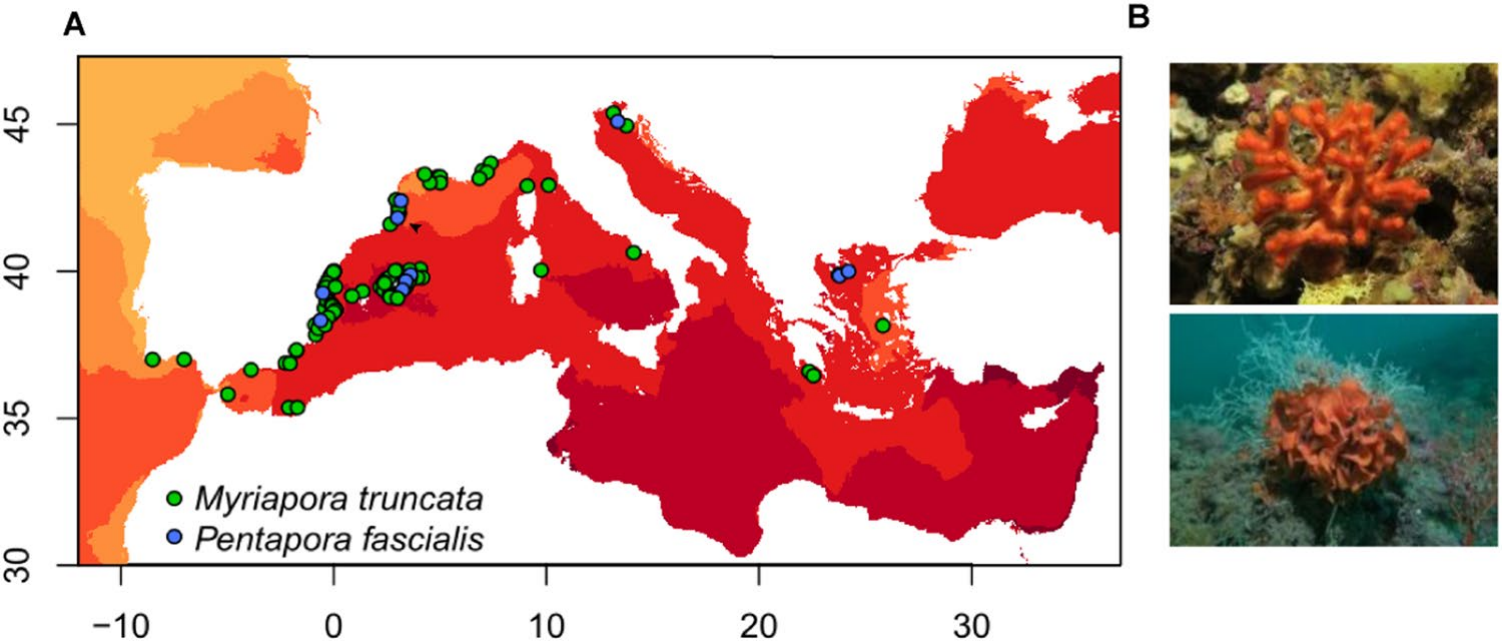
(**A**) The Mediterranean Sea and the study area (arrow) with maximum temperatures of the warmest month (August) represented by a color gradient and the distribution of studied species (occurrence data downloaded from OBIS and GBIF) represented by green and blue dots. (**B**) Model species: *Myriapora truncata* (top) and *Pentapora fascialis* (bottom).

In this study, we focused on bryozoans, abundant colonial filter-feeding invertebrates that inhabit many types of benthic ecosystems, being absent or rare on muddy seabeds. In the Mediterranean Sea, bryozoans are prominent organisms on hard rocky benthic ecosystems, where their colonies can significantly increase the habitat complexity and provide shelter and microhabitats for other organisms^18,24,25^. Because of the fragility of some erect species to physical disturbances, some bryozoans are known as excellent ecological indicators for different stressors, such as storms or physical stress from recreational diving^26–28^. In this study, we selected two model co-occurring species of common and abundant Mediterranean bryozoans, *Myriapora truncata* (Pallas, 1766) and *Pentapora fascialis* (Pallas, 1766), with different distribution patterns at local and regional scales. Despite these two erect calcified species inhabiting similar hard rocky habitats across the Mediterranean, *M*. *truncata* populations are found from 1 m depth in marine caves to 60 m in coralligenous bottoms, reaching 130 m in Tunisian area. In contrast, populations of *P*. *fascialis* are found between 15–100 m depth in rocky bottoms^29^ (Fig. 1). Despite previous field and experimental studies that have studied the effects of warming on Mediterranean bryozoans, most studies have focused on mineralogical, physiological and structural parameters^30,31^. To date, there is a lack of knowledge about the effects of temperature anomalies on demographic parameters of bryozoan populations and our study represents the first attempt to combine results from field and laboratory. Specifically, we combined field data of two erect heavily calcified species during a temperature anomaly in the Mediterranean in the summer of 2015, and the experimental study of the lethal and sublethal effects of thermal stress on both species in aquaria under controlled conditions.

## Results

### Field study

#### Thermal regime in study area

**Figure 2:**
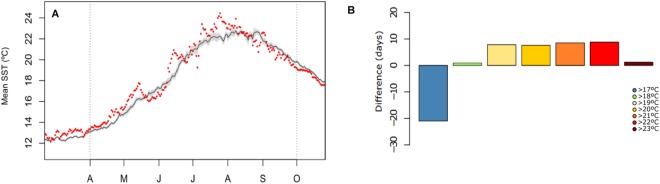
(**A**) Mean annual sea temperature recorded in 2015 (red dots) and the mean temperature recorded from 2005 to 2014 (gray line). (**B**) Difference in the number of days with high temperatures recorded in 2015 and those recorded from the mean of the previous years (2005–2014).

Our results revealed a thermal anomaly in Medes islands during the summer of 2015 when the sea water temperature was higher than the average of the previous years (2005–2014) (19.32 ± 0.22 °C versus 18.99 ± 0.24 °C). Despite this difference was not significant, the maximum temperature reached during summer 2015 was higher than the previous years (24.5 °C versus 22.7 °C), with significantly more days of high temperatures (>22 °C) (p < 0.001) (Fig. 2).

#### Thermal anomaly effects in natural populations

**Figure 3:**
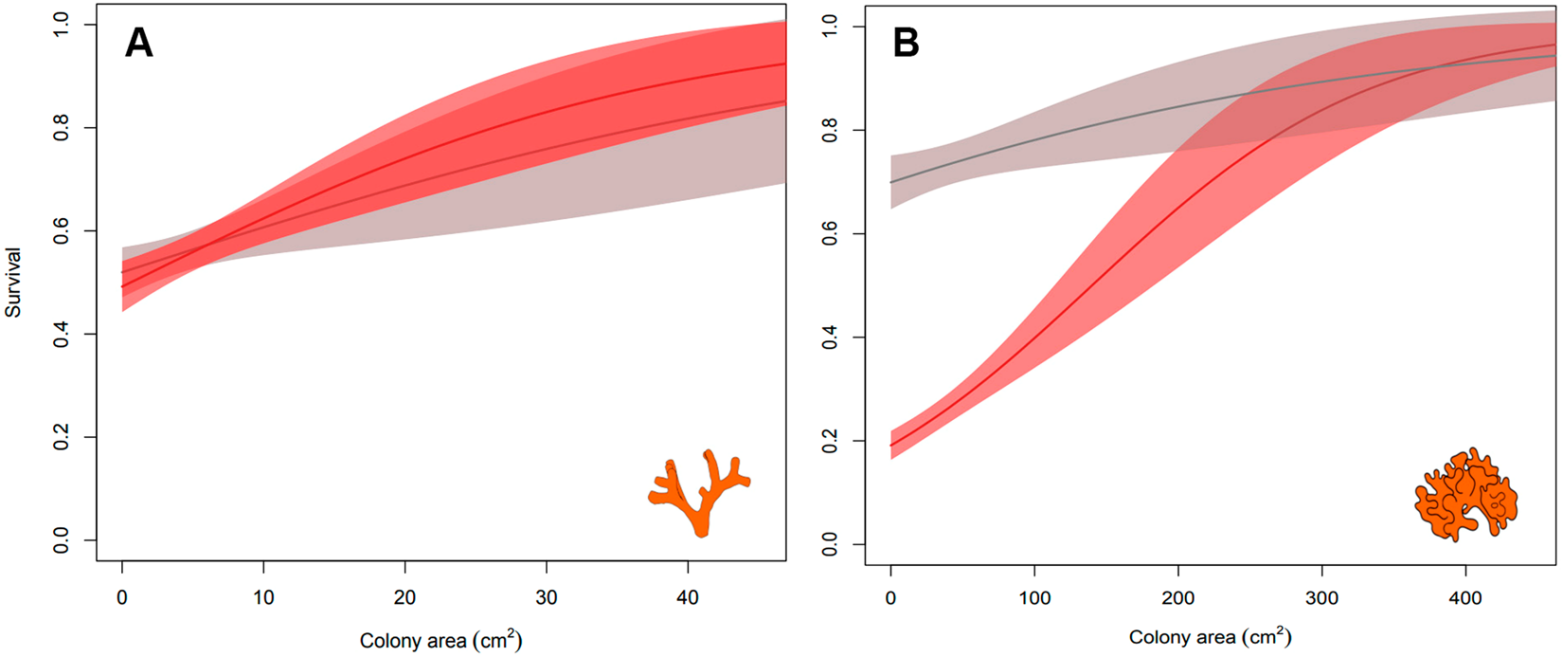
Survival of colonies of *Myriapora truncata* (**A**) and *Pentapora fascialis* (**B**) during winter 2014 (grey) and summer 2015 (red).

We found differences in the survival between the two species in the field after the summer of 2015 (Supplementary Table S1). All colonies of *Myriapora truncata* showed a similar high survival in both studied periods (winter 2014: October 2014 – April 2015; summer 2015: April 2015 – October 2015). In contrast, survival of *Pentapora fascialis* colonies significantly decreased in summer 2015 at all localities (*p* < 0.001), independently of the protection level and habitat type (Fig. 3, Supplementary Table S2). In this period, most *P*. *fasciali*s colonies died or were affected by necrosis (Supplementary Fig. S1). Survival rates were positively related to colony size (*p* < 0.001, Fig. 3, Supplementary Table S2).

### Thermal stress under laboratory conditions

#### Patterns of necrosis

**Figure 4:**
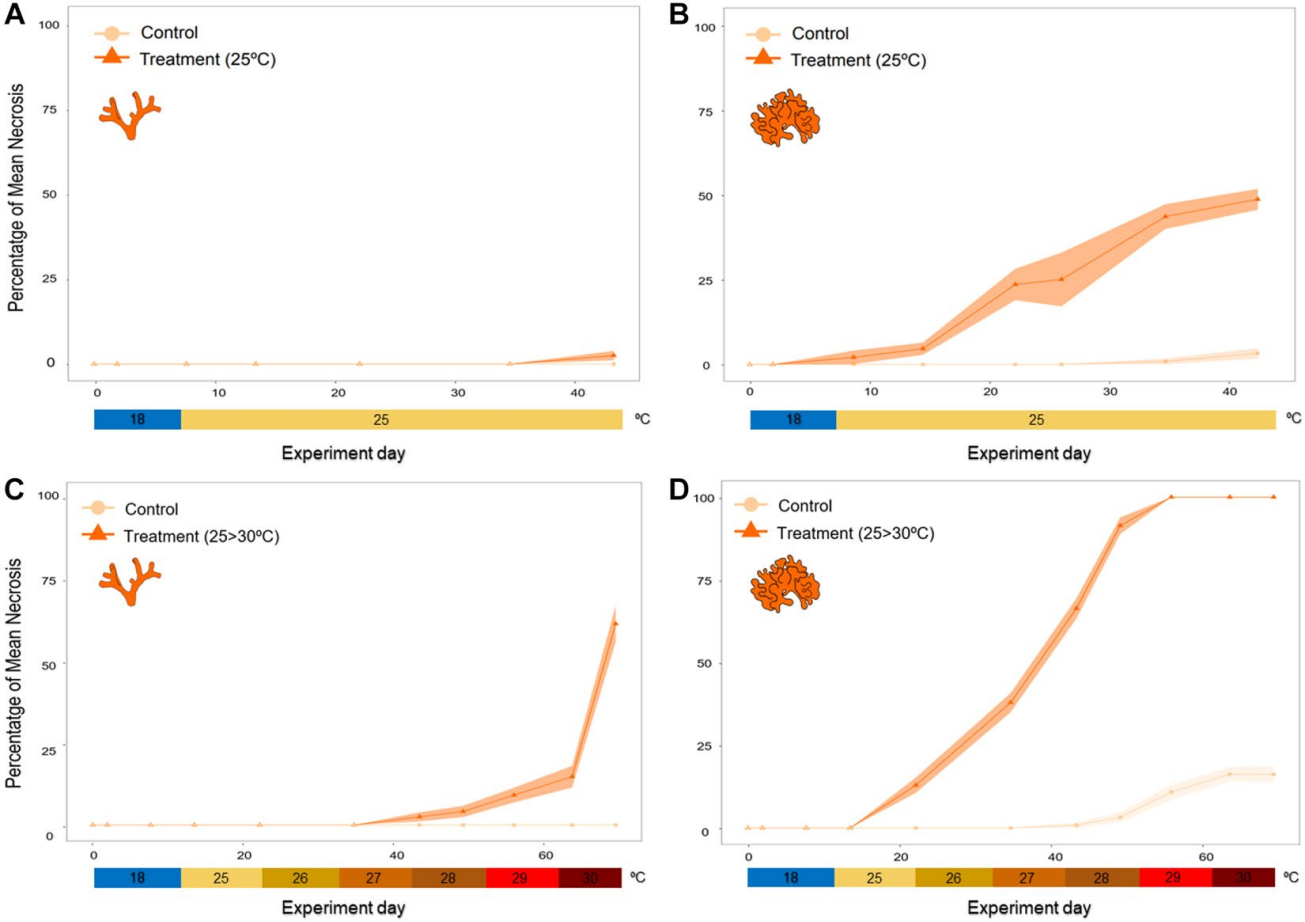
Partial mortality of both species (*Myriapora truncata* (**A,C**); *Pentapora fascialis* (**B, D**)) during thermal stress at 25 °C treatment (**A,B**) and increasing temperature treatment (from 25 °C to 30 °C treatment) (**C,D**).

**Figure 5:**
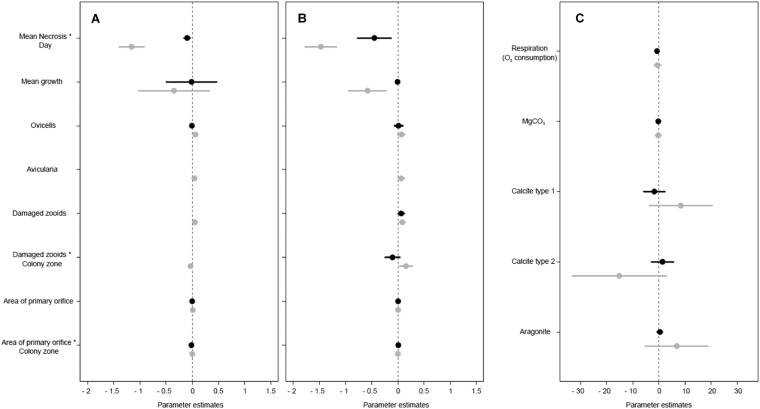
Summary of LM’s parameter coefficients and 95% confidence intervals of studied species (in black, *Myriapora truncata*: in grey, *Pentapora fascialis*) for variables responses between treatments in thermal stress at 25 °C (**A**), increasing temperature experiment (**B**) (see Tables S4, S5) and non-lethal effects variables on thermal stress at 25 °C (**C**).

There were significant differences between the response of species to thermal stress at 25 °C (*p* < 0.001) (Figs 4 and 5, Supplementary Tables S3 and Fig. S2), while in control treatments no sign of mortality was detected for either species. Specifically, colonies of *M*. *truncata* did not show any signals of necrosis until the end of the experiment (44 days), when the colonies started to show small percentages of partial mortality (seen as a loss of coloration, see methodology). In contrast, colonies of *P*. *fascialis* started to exhibit necrosis around day 15. At the end of the experiment, all colonies showed around 50% partial mortality (Figs 4 and 5, Supplementary Table S4).

In the increasing temperature treatment, there were also differences between the responses of the two species to temperature (*p* < 0.001) (Figs 4 and 5, Supplementary Table S3 and Fig. S2). Colonies of *M*. *truncata* started to show necrosis only after 45–50 days when the temperature was 28 °C, and necrosis increased rapidly to 70% when the temperature was raised to 30 °C. In contrast, signs of partial mortality in *P*. *fascialis* colonies were observed after 15–20 days at 25–26 °C, about 20 days earlier and with 2 °C cooler temperature treatment than *M*. *truncata*. After this period, necrosis increased gradually with temperature, showing a 100% of mortality of all colonies when the temperature reached 28 °C after 45 days from the beginning of the experiment (Figs 4 and 5, Supplementary Table S5).

#### Growth rates

**Figure 6:**
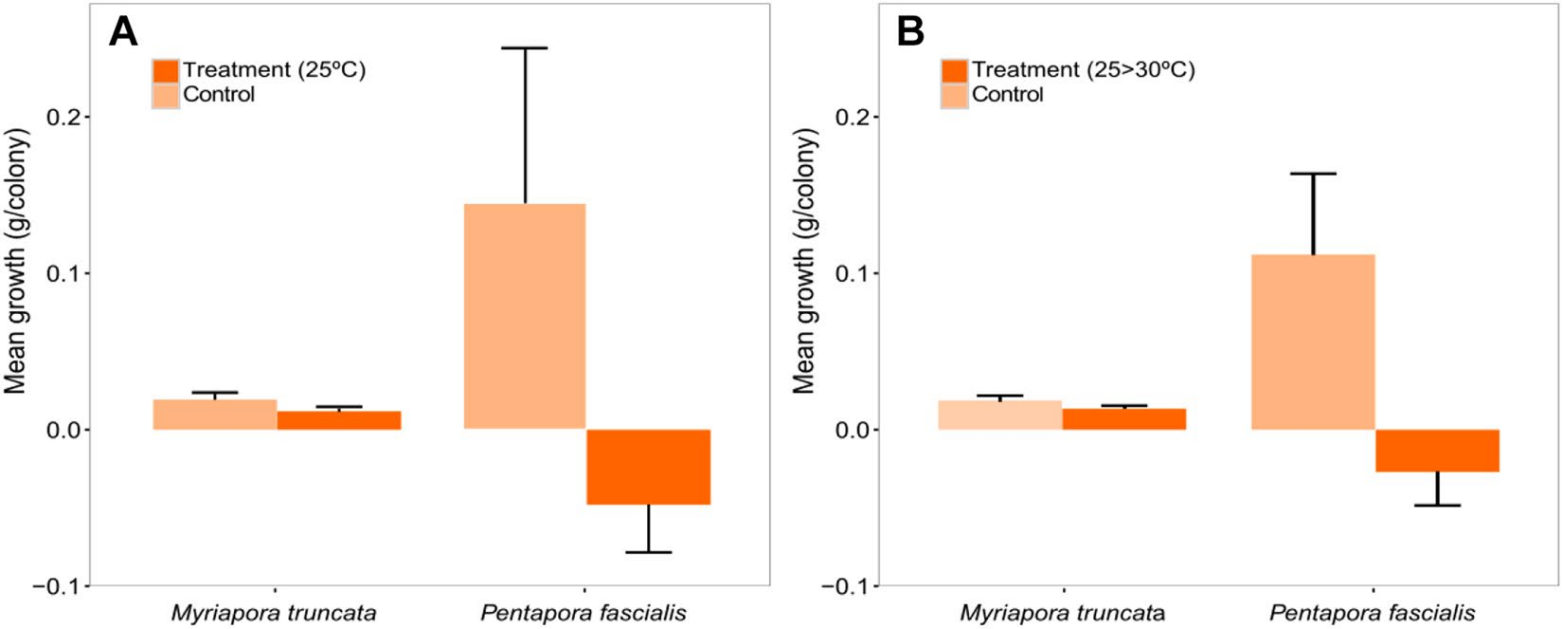
Mean growth (g/colony) between the beginning and the final in thermal stress (25 °C) experiment (**A**) and increasing temperature experiment (**B**) in both species.

Colonies of both species showed higher growth rates in control than in both stress treatments, the differences being larger in *P*. *fascialis* rather than in *M*. *truncata*, but only significant for *P*. *fascialis* in the increasing temperature treatment (*p* < 0.01) (Figs 5 and 6, Supplementary Table S5).

#### Respiration rates

A decrease in oxygen consumption was found in all colonies of both species exposed to temperature treatments. However, this was not significant in both species or between them (Fig. 5, Supplementary Tables S4 and S7).

#### Structural and mineralogical analyses

**Figure 7:**
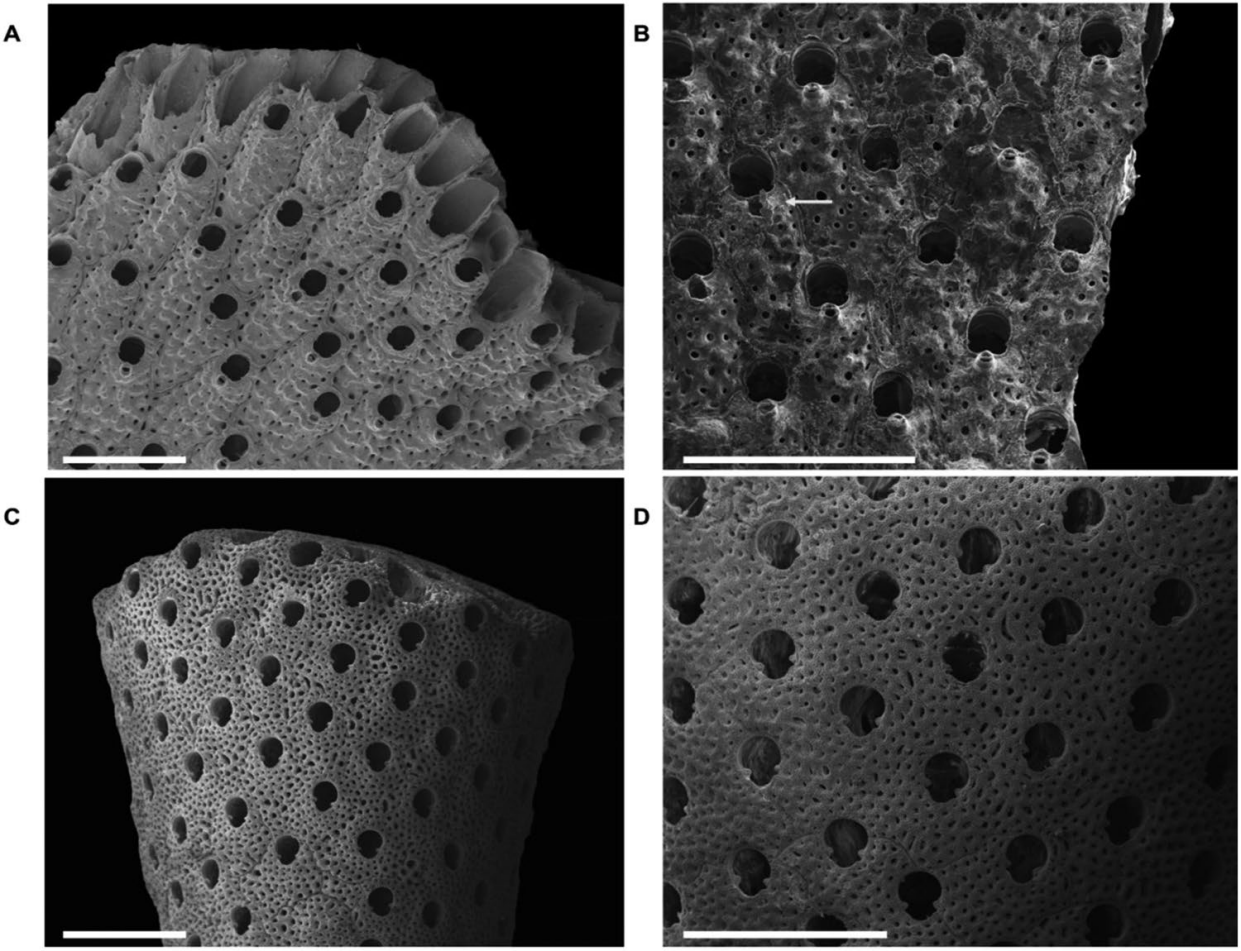
SEM images of *Pentapora fascialis* (**A**,**B**) and *Myriapora truncata* (**C**,**D**). (**A**,**C**) Growing colony edges under control treatment. (**B**) Group of autozooids with some suboral avicularia (arrowed) broken of *P*. *fascialis* under thermal stress treatment. (**D**) Portion of healthy colony of *M*. *truncata* under thermal stress treatment. *Scale bar* 1 mm.

Signs of skeletal damage in the temperature treatments were clearly observed in *P*. *fascialis* but were less evident in *M*. *truncata*. In the first treatment (25 °C), while colonies of *M*. *truncata* showed no signs of damaged zooids, colonies of *P*. *fascialis* showed some damaged zooids (*p* = 0.010) (Figs 5 and 7, Supplementary Tables S3 and S4). Moreover, the mean area of the primary orifice in *P*. *fascialis* was higher in colonies under thermal stress treatment (*p* = 0.010) (Supplementary Table S6).

In the increasing temperature treatment, despite colonies of *M*. *truncata* showed some damaged zooids, there were no significant differences between treatments. In contrast, *P*. *fascialis* also showed damaged zooids in colonies under the increasing temperature treatment, zooids at the distal growth tips of the colonies being most affected (*p* = 0.010) (Fig. 5, Supplementary Table S5). Moreover, as in thermal stress experiment, colonies of this species showed higher values for the mean area of the primary orifice (*p* = 0.044) (Supplementary Table S6). The other structural variables showed no trends between treatments and experiments (Supplementary Tables S3 and S4).

Mineralogical analyses did not show clear differences between treatments; however, we observed higher values of Mg in both species and of aragonite in *P*. *fascialis* when the colonies were exposed to high temperatures (Fig. 5, Supplementary Tables S4 and S7).

## Discussion

Assessing the thermal vulnerability of under-studied but abundant groups of organisms which are important members of many benthic communities such as bryozoans is vital to better understand how the distribution and structure of benthic communities will change under a warming ocean. Our results provide novel insights into the contrasting thermal vulnerability of two abundant and sympatric bryozoan species, highlighting the importance of studying species-specific responses.

Survival patterns in the field revealed a clearly contrasting vulnerability of each species to a thermal anomaly that occurred during summer 2015, showing a clear impact on populations of *Pentapora fascialis* (mainly affecting small-sized colonies), and negligible effects on *Myriapora truncata*. This result agrees with data from previous mass mortality events in the Mediterranean, which revealed *P*. *fascialis* to be among the affected species together with gorgonians, corals or sponges^5,6,32^. However, these previous studies did not detect differences between these two co-occurring bryozoan species. In agreement with field observations, aquarium experiments revealed differences in the vulnerability to thermal stress between the studied species, where *M*. *truncata* displayed a higher resistance to a wide range of thermal stress conditions. The maximum registered temperatures in our study area are 23–24 °C and an increment of 1–2 °C is predicted for the end of this century^4^, suggesting that the ongoing warming trend may be critical for populations of *P*. *fascialis* in the future. Our findings also showed the importance of linking observational and experimental studies to understand the effects of climate change and its consequences for marine species inhabiting in similar habitats.

Beyond the lethal effects of thermal stress, our study also demonstrates other non-lethal effects of warming in *P*. *fascialis*, and in *M*. *truncata* to a less extent. On one hand, our results showed a decrease of growth caused by warming, which has been described in other studies focused on bryozoans^30,33,34^. On the other hand, in both species we registered a decreasing trend of respiration rates in colonies submitted to thermal stress, which suggest sublethal effects on their metabolic activity under thermal stress, in spite of the absence of necrosis in *M*. *truncata*. Moreover, structural analyses confirmed the negative effects of temperature on *P*. *fascialis*. In particular, analyses on Scanning Electron Microscope (SEM) showed a major proportion of damaged zooids in colonies exposed to temperature treatments in this species. Similarly, mean area of primary orifices was higher in temperature treatments, which may indicate that this orifice was broken and was registered as damaged zooids. These results suggest a dissolution or removing of the skeleton around the orifice increasing its area. Previous studies showed that temperature has effects on zooid size, skeletal growth, biomineral deposition and carbonate production on many species of bryozoans^35,36^. However, some of them showed that only a positive interaction between temperature and pH caused the corrosion of the *M*. *truncata* skeleton^31,37^ highlighting the need to improve our understanding of the mechanisms behind the physiological responses of *P*. *fascialis* to thermal stress. On the other way, although the differences were not significant, we observed higher values of Mg in both species and of aragonite in *P*. *fascialis* respectively when the colonies were exposed to high temperatures. These findings agree with previous studies in other groups and other bryozoan species^38,39^. However, our results should be taken cautiously given the reduced sample size and the short-term exposures to these conditions.

Life history traits may also influence the response of species under climate change scenario^40^. Despite a general lack of information about the life history traits and population dynamics of bryozoans, there is evidence that *P*. *fascialis* grows faster than *M*. *truncata*^29,41^. The high vulnerability showed by *P*. *fascialis* is in accordance with the idea that species with faster dynamics are more vulnerable to environmental changes^42^. Future studies should explore the relationship between life-history traits and vulnerability in bryozoans and other temperate invertebrates.

The distributional patterns of species reflect their realized niche and environmental tolerances^43^. Both bryozoan species inhabit similar habitats across the Mediterranean, from shallow hard-rocky bottoms to coralligenous assemblages, however it is known that populations of *M*. *truncata* can be shallower than *P*. *fascialis*^29^. In this way, we hypothesize that in the areas where the temperature is warmer, *P*. *fascialis* may habit deeper where the temperature is not so high, contrary to populations of *M*. *truncata*. Accordingly, many studies showed that the coexistence of similar species involves divergences in some ecological aspect, as temperature tolerance, to adapt to different parts of the environmental gradient^44,45^ which agrees with our results. Nevertheless, it is crucial to explore the relationship between distribution patterns and environmental variables to accurately model their ecological niches. However, it is important to take into account the limitations of working with open databases. In our study, we found that due to the actual disagreement about whether *P*. *fascialis* and *P*. *foliacea* are separate species, all registers in north-Atlantic bryozoan *P*. *foliacea* were recorded as. *P*. *fascialis*^46^. This highlights the importance to complement the use of open databases such as OBIS or GBIF with the taxonomic and ecological knowledge of the target species to avoid errors in the interpretation of distributional patterns.

To sum up, to understand the future of ectotherms under climate change it is necessary a better understanding of how species diverge in climatic niches to forecast the response of species to warmer conditions and which are in risk of local extinction^15^. Future studies should integrate a better knowledge of ecological niches, demographic processes and physiological responses to predict the future of vulnerable populations in a changing world.

## Methods

### Study species and study area

Populations of both species were monitored in 7 locations at 18–25 m depth in the Montgrí, Medes Islands and Baix Ter Natural Park in the North-Western Mediterranean (Fig. 1, Supplementary Fig. S3). We monitored all the locations every 6-months through one year (October 2014, April 2015 and October 2015) to describe bryozoan population dynamics between the summer and winter periods. Selected locations were classified by protection level (unfrequented *versus* highly frequented by recreational SCUBA divers) and by type of habitat (hard rock bottoms or coralligenous walls). In each location, we installed a permanent transect of 10 m^2^ where colonies of *P*. *fascialis* and *M*. *truncata* were individually identified, and their heights, diameters, and degrees of exposure were measured *in situ* by SCUBA diving following similar procedures^26^. Moreover, we photographed all the colonies to obtain other parameters such as colony area through image analyses (Photoshop CC2017). Through demographic analyses, we obtained parameters such as survival and recruitment between the studied periods (winter 2014: October 2014 – April 2015; summer 2015: April 2015 – October 2015). In modular marine species, the age of individual colonies is hard to assess and life-history traits as survival or growth are often modeled as a function of colony size^47^. Thus, we fitted a set of regression models of survival and colony size data to explore the potential effects of multiple factors (season, habitat type, etc.).

On the other hand, local thermal regimes during the monitoring period were quantified by high-resolution hourly temperature recordings at 20 m depth obtained from the T-MedNet platform (http://www.t-mednet.org/).

### Experimental thermal stress study

#### Sample collection and experimental design

To explore differential responses to thermal stress between the two bryozoan species, we studied them under experimentally controlled conditions. One fragment of 3–5 cm height from 90 healthy colonies of each species were sampled at the same depth (approximately 20 m) in Medes Islands during October 2016 and transported in aerated seawater to the Experimental Aquarium Facilities of the Institute of Marine Sciences in Barcelona (in less than 24 hours). All the colonies were set in aquaria tanks (approximately 48 l volume), continuously supplied with seawater (salinity 38 ppm) and a current of flow rate around 60 l h^−1^ generated by a submersible pump and were subjected to an acclimation period of 7 days at 18 °C^21,48^. The colonies were fed three times per week with 3 ml of a liquid mixture of particles between 10 to 450 μm in size (Benthos Nutrition Marine Active Supplement, Maim, Vic, Spain) in each aquarium.

We designed 3 different treatments, one control, and 2 different temperature treatments; each utilized of three tanks (replicates) containing 10 colonies from each species. In the control treatment, seawater temperature was maintained by a continuous flow-through of new water at 18 °C. In the temperature treatments, the seawater was heated with submersible resistance heaters regulated by temperature controllers (Aqua Medic T controllers). The first temperature treatment simulated a relatively large period of high temperatures subjecting the colonies to 25 °C for a period of 44 days. This temperature has been recorded in several mass mortality events and identified as a critical threshold for several Mediterranean invertebrate species^22,48,49^. In contrast, the second temperature treatment was subjected to a sequential increase of temperature from 25 °C to 30 °C, to investigate thermotolerance features of both species and detect the maximum critical temperature. Specifically, the temperature was increased firstly to 25 °C and, from there it was increased 1 °C every 5–7 days until reaching 30 °C when the experiment finished after 72 days. This methodology was previously used in several studies and demonstrated to be effective to study thermotolerance ranks in benthic species^20,21^.

#### Variables studied

We carried out photographic monitoring of all the colonies at regular intervals of 3–6 days and we quantified the proportion of necrosis (dead tissue) expressed as % of the total area through image analysis (Photoshop CC2017). Necrosis was expressed as the proportion of the areas presenting a loss of colony coloration derived from the partial or total lost of living tissue covering the skeleton^6,41,50^, following previous studies on corals or gorgonians^20,21,51^. Necrosis rates were estimated in 10% intervals, and we considered a colony to be affected by partial mortality when it showed recent necrosis over 10% of tissue^21^. On the other hand, to obtain the mean growth of the colonies, all of them were weighted at the beginning and at the end of the experiment using the buoyant weight technique^52^.

We also tested whether thermal stress may drive physiological non-lethal effects by comparing respiration rates between the temperature treatment at 25 °C and the control. To achieve this, 6 healthy specimens for each treatment and species were incubated for 12 hours in individual chambers (130 ml in volume) that were completely filled with 50 μm pre-filtered seawater (without any air space) and hermetically closed, according to the standardized protocol^53^. Moreover, 6 chambers, filled with pre-filtered sea water without any bryozoan, were used as controls. Chambers were maintained at a constant temperature in a water bath (18 and 25 °C, respectively), and a Teflon-coated magnetic stirrer ensured water movement inside each incubation chamber. Oxygen concentration in each chamber was recorded at the beginning and end of the incubation, using an optode sensor (YSI ProODO Optical Dissolved Oxygen meter, precision 0.2 mg L^−1^). Variation in the oxygen concentration measured from the control chambers was subtracted from those measured in the bryozoan chambers, and oxygen consumptions were derived from the dissolved oxygen over the incubation and were normalized by colony weight.

To perform skeletal structure and mineralogical analysis, at the end of the experiment we collected from all treatments different fragments from two colony areas (proximal and distal zones) from each specimen in both species. The samples were stored and prepared to be observed with a Scanning Electron Microscope (SEM). Specifically, we selected 3 replicates of 1 × 1 mm^2^ per colony and zone, and we registered structural variables such as the density of zooids, ovicells, avicularia, damaged or broken zooids, and the mean area of the primary orifice^29,37^. Moreover, to evaluate the non-lethal effects in the skeletal content, we quantified calcite and Mg content of the calcite (type 1: low-magnesium calcite; type 2: high-magnesium calcite)^54^ and aragonite on colonies subjected to thermal stress experiment (25 °C) at the end of the experiment. We performed mineralogical analyses cutting 3 replicates (2 × 2 mm^2^) from the growing edge following previously described methodologies^55^. The pieces with 10 grains of pure halite (NaCl) as an internal standard were powdered using a quartz pestle and mortar. The samples were sandwiched between films of polyester of 3.6 microns of thickness. X-ray powder diffraction (XRD) was performed on PANalytical X’Pert PRO MPD powder diffractometer (240 mm goniometer radius) equipped with a PIXcel detector and operating with a Cu Kα (λ = 1.5418 Å) radiation source generated at a voltage of 45 kV and a current of 40 mA at the Scientific and Technological Centers of the UB (CCiT-UB). An angular range of 4 to 65° 2θ was measured with a step size of 0.026° and a 200 s counting time per step. Soller and incident slits were set to 0.04 rad and 0.7 mm, respectively. To determine the proportions of aragonite and calcite, peak intensities were fitted to standard patterns generated from 100% aragonite and 100% calcite. The wt% MgCO_3_ in calcite was calculated by measuring the position of the d104 peak, assuming a linear interpolation between CaCO_3_ and MgCO_3_^56^ and recalibrated for the specific machine used. A linear trend of d104 versus mol% MgCO_3_ can be observed in the range between 0 and 17 mol% MgCO_3_^57^. All data of this study fall into this range.

### Statistical analysis

To reveal differences between maximum temperatures between our study period and the previous years we used Linear Models (LM). Moreover, to analyze the results of field study we used General mixed models (GLM) fitting binomial distributions of the errors to test the relation between the survival and census period (time), locality, protection level, habitat type, and colony-size (colony area). On the other hand, we used LM’s to test for differences between aquaria treatments and species in several parameters that indicate lethal and sub-lethal physiological effects: percentage of necrosis, growth rate, respiration rates, skeletal structure (as density of zooids, ovicells, avicularia, damaged or broken zooids, and the medium area of primary orifice) and mineralogy (Mg content, calcite and aragonite). All statistical analysis and graphics were produced using R version 3.1.2^58^ (R Core Developer Team 2014).

## Electronic supplementary material

Supplementary Information

## Data Availability

The datasets generated and/or analyzed during the current study are available from the corresponding author on reasonable request.
