# Supplementary material for: Divergent responses to warming of two common co-occurring Mediterranean bryozoans
Source: Sci Rep. 2018 Nov 29;8:17455. doi: 10.1038/s41598-018-36094-9 (PMC6265274; doi:10.1038/s41598-018-36094-9)

Divergent responses to warming of two common co-occurring Mediterranean bryozoans

Marta Pagès-Escolà[1*], Bernat Hereu[1], Joaquim Garrabou[2], Ignasi Montero-Serra[1,2], Andrea Gori[2], Daniel Gómez-Gras[2], Blanca Figuerola[3], Cristina Linares[1]

[1] Department of Evolutionary Biology, Ecology and Environmental Sciences, Institut de Recerca de la Biodiversitat (IRBIO), University of Barcelona, Av. Diagonal 643, 08028 Barcelona, Spain

[2] Institute of Marine Sciences, ICM-CSIC, Pg. Marítim de la Barceloneta 37-49, 08003 Barcelona, Spain

[3] Smithsonian Tropical Research Institute, P.O. Box 0843-03092, Balboa, Republic of Panama

*Corresponding author: mpagesescola@gmail.com

Supplementary information

Supplementary Table S1. Statistical results of Generalized Mixed Models (GLMs) of mass mortality event in the field indicating each model in each case with the response variable (S = survival) and the interaction between Species and the studied variables

Supplementary Table S2. Statistical results of Generalized Mixed Models (GLMs) of mass mortality event in the field both species indicating in each case the response variable (Survival) and the studied variables.

Supplementary Table S3. Statistical results of Linear Models (LMs) in two experiments between the studied variables and the interaction of treatments and species.

Supplementary Table S4. Statistical results of Linear Models (LMs) in Thermal stress experiment (25ºC) between the studied variables and the two treatments.

Supplementary Table S5. Statistical results of Linear Models (LMs) in the increasing temperature experiment between the studied variables and the two treatments.

Supplementary Table S6. SEM variables (mean ± SE) of colonies between species and treatment in thermal stress experiment and increasing temperature experiment.

Supplementary Table S7. Summary of non-lethal effects on oxygen consumption and mineralogical variables between species in thermal stress experiment (25ºC).

Supplementary Figure S1. Demographic analyses of monitored populations during the mass mortality event.

Supplementary Figure S2. Photographic analyses of thermal stress experiment at 25ºC (A) and increasing temperature experiment.

Supplementary Figure S3. Study area and location of monitored localities within the Medes Islands Marine Reserve (points).

Supplementary Table S1. Statistical results of Generalized Mixed Models (GLMs) of mass mortality event in the field indicating each model in each case with the response variable (S = survival) and the interaction between Species and the studied variables

**Models**	**Variables**	**Coefficients**				**AICc**
		Estimate	Std. Error	*z value*	*p*	
Survival ~ Species*Season	Intercept	-1.2566	0.10785	-11.681	**<0.001**	2042.059
	Species	0.2885	0.1495	1.930	0.0535	
	Season	0.09032	0.1503	0.601	0.5479	
	Species*Season	-0.7869	0.222	3.539	**<0.001**	
Survival ~ Species*Habitat	Intercept	-1.2066	0.0755	-15.984	**<0.001**	2032.883
	Species	0.0708	0.1118	0.634	0.526	
	Habitat	-0.4028	0.7782	-0.518	0.605	
	Species*Habitat	-1.2023	0.8635	-1.392	0.164	
Survival ~ Species* Protection	Intercept	-1.0746	0.0825	-13.023	**<0.001**	2030.34
	Species	0.00102	0.1228	0.008	0.9933	
	Protection	-0.7018	0.2055	-3.415	**<0.001**	
	Species*Protection	-0.07299	0.2819	-0.259	0.7956	
Survival ~ Species*Colony Area	Intercept	-1.8278	0.10519	-17.376	**<0.001**	1496.911
	Species	-0.0317	0.15564	-0.204	0.838	
	Colony Area	0.2607	0.02610	9.988	**<0.001**	
	Season*Colony Area	-0.2367	0.02630	-9.001	**<0.001**	

Supplementary Table S2. Statistical results of Generalized Mixed Models (GLMs) of mass mortality event in the field both species indicating in each case the response variable (Survival) and the studied variables.

**Models**	**Variables**	***Myriapora truncata***					***Pentapora fascialis***				
		**Coefficients**				**AICc**	**Coefficients**				**AICc**
		Estimate	Std. Error	*z value*	*p*		Estimate	Std. Error	Estimate	Std. Error	
Survival ~ Season	Intercept	-1.2566	0.1076	-11.681	**<0.001**	1083.248	-0.9681	0.1038	-9.324	**<0.001**	958.8
	Season	0.0903	0.1503	0.601	0.548		-0.6966	0.1639	-4.251	**<0.001**	
Survival ~ Habitat	Intercept	-1.2066	0.0755	-15.984	**<0.001**	1083.317	-1.1357	0.0825	-13.762	**<0.001**	949.57
	Habitat	-0.4028	0.7783	-0.518	0.605		-1.6051	0.374	-4.292	**<0.001**	
Survival ~ Protection	Intercept	-1.0746	0.0825	-13.023	**<0.001**	1070.655	-1.0736	0.0910	-11.797	**<0.001**	959.69
	Protection	-0.7018	0.2055	-3.415	**<0.001**		-0.7748	0.1929	-4.016	**<0.001**	
Survival ~ Colony Area	Intercept	-1.8278	0.1052	-17.376	**<0.001**	815.997	-1.8595	0.11471	-16.212	**<0.001**	681.51
	Colony Area	0.2607	0.0261	9.988	**<0.001**		0.02404	0.00316	7.608	**<0.001**	
Survival ~ Season*Habitat	Intercept	-1.2411	0.1077	-11.517	**<0.001**	1083.965	-0.7501	0.1072	-6.994	**<0.001**	915.45
	Season	0.0684	0.1510	0.453	0.615		-0.8684	0.1722	-5.043	**<0.001**	
	Habitat	-13.3249	360.3785	-0.037	0.971		-16.8160	486.9721	-0.035	0.972	
	Season*Habitat	13.8045	360.3795	0.038	0.969		16.4535	486.9723	0.034	0.973	
Survival ~ Season*Protection	Intercept	-1.1795	0.1199	-9.840	**<0.001**	1071.403	-0.7503	0.1201	-6.245	**<0.001**	944.76
	Season	0.2046	0.1654	1.237	0.2161		-0.7083	0.1870	-3.787	**<0.001**	
	Protection	-0.3681	0.2739	-1.344	0.1790		-0.7874	0.2413	-3.184	**<0.01**	
	Season*Protection	-0.7072	0.4181	-1.691	0.0908		-0.0053	0.4008	-0.013	0.9895	
Survival ~ Season*Colony Area	Intercept	-1.863	0.1445	-12.896	**<0.001**	819.286	-2.0800	0.1673	-12.434	**<0.001**	641.69
	Season	0.0765	0.2108	0.363	0.716		0.2132	0.2409	0.885	0.376	
	Colony Area	0.2681	0.0386	6.935	**<0.001**		0.0639	0.0086	7.442	**<0.001**	
	Season*Colony Area	-0.0147	0.0524	-0.281	0.779		-0.0495	0.0092	-5.396	**<0.001**	

Supplementary Table S3. Statistical results of Linear Models (LMs) in two experiments between the studied variables and the interaction of treatments and species.

Models	Variables	Thermal experiment (25ºC)					Increasing temperature experiment (25>30ºC)				
		Coefficients					Coefficients				
		Estimate	Std. Error	t value	*p*	Adjusted R-squared	Estimate	Std. Error	t value	*p*	Adjusted R-squared
Mean necrosis ~Treatment * Sp * Day	Intercept	-0.93167	1.5334	-0.608	0.5492	0.9547	-7.2063	4.9068	-1.469	0.1506	0.8998
	Treatment	0.93167	2.1684	0.430	0.6713		7.2063	6.9393	1.038	0.3059	
	Sp	-4.3421	2.1684	-2.002	0.0567		-4.2691	6.9393	-0.615	0.5422	
	Day	0.0981	0.0626	1.568	0.1299		0.4576	0.1162	3.938	**<0.001**	
	Treatment * Sp	3.8121	3.0667	1.243	0.2258		1.3072	9.8136	0.133	0.8947	
	Treatment * Day	-0.0981	0.0885	-1.109	0.2785		-0.4576	0.1643	-2.785	**<0.01**	
	Sp * Day	1.1169	0.0885	12.617	**<0.001**		1.2354	0.1643	7.518	**<0.001**	
	Treatment * Sp * Day	-1.0631	0.1252	-8.492	**<0.001**		-1.0216	0.2324	-4.396	**<0.001**	
Mean growth ~ Treatment * Sp	Intercept	-0.42915	0.2141	-2.004	**0.0473**	0.01288	0.0265	0.0967	0.274	0.7846	0.169
	Treatment	-0.01407	0.3027	-0.046	0.9630		-0.0076	0.1297	-0.059	0.9531	
	Sp	0.23303	0.3027	0.770	0.4431		0.5164	0.1368	3.775	**<0.001**	
	Treatment*sp	-0.34465	0.4281	-0.805	0.4225		-0.5735	0.1835	-3.125	**<0.01**	
O_2 consumption ~ Treatment * Sp	Intercept	31.8533	13.4068	2.376	**0.0448**		-	-	-	-	-
	Treatment	-92.33	0.6155	-1.500	0.1720		-	-	-	-	
	Sp	-4.7724	18.9601	-2.525	0.8076		-	-	-	-	
	Treatment * Sp	0.2448	0.8704	0.281	0.7857		-	-	-	-	
Density ovicells ~ Treatment * Sp	Intercept	0.0074	0.0114	0.645	0.5194	0.03511	0.0901	0.0273	3.299	**<0.01**	0.008
	Treatment	-0.0074	0.0164	-0.451	0.6525		0.0118	0.0409	0.290	0.7719	
	Sp	-0.0074	0.0165	-0.450	0.6531		-0.0149	0.0378	-0.396	0.6925	
	Treatment*Sp	0.0716	0.0231	3.094	**<0.01**		0.0570	0.0564	1.012	0.3122	

Damaged zooids ~Treatment * Sp	Intercept	5.533^{-16}	0.0096	0	1	0.03186	0.0491	0.0227	2.160	**0.0313**	0.0167
	Treatment	-1.019^{-15}	0.0138	0	1		0.0630	0.0341	1.848	0.0652	
	Sp	-4.67^{-16}	0.0138	0	1		-0.0266	0.0315	-0.844	0.3989	
	Treatment * Sp	0.05	0.0194	2.567	**0.0105**		0.0224	0.047	0.478	0.6326	
Area of primary orifice ~ Treatment * Sp	Intercept	0.0361	0.0024	14.748	**<0.001**	0.0941	0.0358	0.0016	21.961	**<0.001**	0.094
	Treatment	-0.0021	0.0035	-0.618	0.5370		0.0041	0.0024	1.674	0.0949	
	Sp	-0.0143	0.0035	-4.076	**<0.001**		-0.0112	0.0022	-4.971	**<0.001**	
	Treatment * Sp	0.0114	0.0049	2.310	**0.0213**		-0.0002	0.0034	-0.075	0.9403	
$MgCO_3$ ~Treatment * Sp	Intercept	8.3305	0.3284	25.366	**<0.001**	0.166	-	-	-	-	-
	Treatment	-26.45	0.4644	-0.569	0.575		-	-	-	-	
	Sp	0.5855	0.4644	1.261	0.222		-	-	-	-	
	Treatment * Sp	0.511	0.6568	0.839	0.411		-	-	-	-	
Calcite 1~Treatment * Sp	Intercept	73.242	3.2	22.885	**<0.001**	0.4199	-	-	-	-	-
	Treatment	-1.759	4.526	-0.389	0.7016		-	-	-	-	
	Sp	-17.894	4.526	-3.953	**<0.001**		-	-	-	-	
	Treatment * Sp	10.105	6.401	1.579	0.13		-	-	-	-	
Calcite 2~Treatment * Sp	Intercept	26.062	5.020	5.192	**<0.001**	0.4129	-	-	-	-	-
	Treatment	1.363	7.099	0.192	0.850		-	-	-	-	
	Sp	-10.917	7.099	-1.538	0.140		-	-	-	-	
	Treatment * Sp	-16.509	10.040	-1.644	0.116		-	-	-	-	
Aragonite ~Treatment * Sp	Intercept	0.6956	3.2421	0.215	0.832	0.8079	-	-	-	-	-
	Treatment	0.3962	4.5850	0.086	0.932		-	-	-	-	
	Sp	28.8102	4.5850	6.284	**<0.001**		-	-	-	-	
	Treatment * Sp	6.4036	6.4841	0.988	0.335		-	-	-	-	

Supplementary Table S4. Statistical results of Linear Models (LMs) in Thermal stress experiment (25ºC) between the studied variables and the two treatments.

Models	Variables	***Myriapora truncata***					***Pentapora fascialis***				
		**Coefficients**					**Coefficients**				
		Estimate	Std. Error	t value	*p*	Adjusted R-squared	Estimate	Std. Error	t value	*p*	Adjusted R-squared
Necrosis ~Treatment*Day	Intercept	-0.93167	0.5417	-1.720	0.111	0.5856	-5.2738	2.0997	-2.512	0.0273	0.952
	Treatment	0.93167	0.7660	1.216	0.247		4.7437	2.9694	1.598	0.1361	
	Day	0.09816	0.0221	4.439	**<0.001**		1.2151	0.0857	14.176	**<0.001**	
	Treatment*Day	-0.09816	0.0312	-3.139	**<0.01**		-1.1613	0.1212	-9.580	**<0.001**	
Mean growth ~ Treatment	Intercept	-0.42915	0.1754	-2.466	0.0175	**0.01719**	-1.1961	0.2468	-0.795	0.430	0.01789
	Treatment	-0.01407	0.2481	-0.057	0.9550		-0.3587	0.3490	-1.028	0.308	
O_2 consumption ~ Treatment	Intercept	31.8533	9.5672	3.329	**0.0291**	0.4061	27.0810	16.363	1.654	0.173	-0.0383
	Treatment	-0.9233	0.4392	-2.102	0.1034		-0.6786	0.7515	-0.903	0.418	
Density ovicells ~ Treatment	Intercept	0.0074	0.0053	1.398	0.163	0.0036	-6.104^{-17}	0.0157	0	1	0.0281
	Treatment	-0.0074	0.0076	-0.977	0.329		0.0642	0.0217	2.954	**<0.01**	
Density avicularia ~ Treatment	Intercept	-	-	-	-	-	0.02344	0.0189	1.237	0.2170	0.012
	Treatment	-	-	-	-		0.04399	0.0262	1.831	0.0682	
Damaged zooids ~Treatment	Intercept	-	-	-	-	-	-1.628^{-16}	0.0139	0	1	0.0208
	Treatment	-	-	-	-		0.05	0.0193	2.586	**0.0102**	
Damaged zooids ~Treatment* Colony zone	Intercept	-	-	-	-	-	2.768^{-16}	0.0096	0	1	0.0195
	Treatment	-	-	-	-		0.0428	0.0135	3.163	**<0.01**	
	Colony zone	-	-	-	-		-3.614^{17}	0.0139	0	1	
	Treatment* Colony zone	-	-	-	-		-0.0351	0.0196	-1.790	0.0739	
Area of primary orifice ~ Treatment	Intercept	0.0636	0.0023	15.190	**<0.001**	0.00154	0.0218	0.0025	8.436	**<0.001**	0.0208
	Treatment	-0.00216	0.0034	-0.636	0.525		0.0092	0.0035	2.584	**0.0103**	
Area of primary orifice ~ Treatment * Colony zone	Intercept	0.0292	0.0032	9.116	**<0.001**	0.0273	0.0206	0.0036	5.715	**<0.001**	0.0142
	Treatment	0.0055	0.0046	1.183	0.2384		0.0105	0.0049	2.130	**0.0341**	
	Colony zone	0.0147	0.0047	3.140	**<0.01**		0.0024	0.0052	0.462	0.6443	
	Treatment* Colony zone	-0.0164	0.0067	-2.437	**0.0155**		-0.0026	0.002	-0.368	0.7132	
$MgCO_3$ ~Treatment	Intercept	8.3305	0.2215	37.613	**<0.001**	-0.0268	8.916	0.4082	21.840	**<0.001**	-0.0735
	Treatment	-0.2645	0.3132	-0.844	0.418		0.2866	0.5773	0.496	0.63	
Calcite 1~Treatment	Intercept	73.242	1.419	51.627	**<0.001**	-0.0214	55.348	4.298	12.878	**<0.001**	0.0745
	Treatment	-1.759	2.006	-0.877	0.401		8.346	6.078	1.373	0.2	
Calcite 2~Treatment	Intercept	26.062	1.516	17.190	**<0.001**	-0.0573	15.146	6.935	2.184	0.0539	0.1118

	Treatment	1.363	2.144	0.636	0.539		-15.146	9.808	-1.544	0.1536	
Aragonite ~Treatment	Intercept	0.6956	0.3610	1.927	0.0829	-0.0375	29.506	4.571	6.455	<**0.001**	0.0095
	Treatment	0.3962	0.5106	0.776	0.4558		6.8	6.464	1.052	0.318	

Supplementary Table S5. Statistical results of Linear Models (LMs) in the increasing temperature experiment between the studied variables and the two treatments.

**Models**	**Variables**	***Myriapora truncata***					***Pentapora fascialis***				
		**Coefficients**					**Coefficients**				
		Estimate	Std. Error	t value	*p*	Adjusted R-squared	Estimate	Std. Error	t value	*p*	Adjusted R-squared
Necrosis (%) ~Treatment*Day	Intercept	-7.2063	5.2819	-1.364	**0.1893**	0.4858	-11.4754	4.5006	-2.550	0.0201	0.9473
	Treatment	7.2063	7.4697	0.965	**0.3475**		8.5135	6.3648	1.338	0.1977	
	Day	0.4576	0.1251	3.659	**<0.01**		1.6929	0.1066	15.886	**<0.001**	
	Treatment*Day	-0.4576	0.1769	-2.587	**0.0186**		-1.4792	0.1507	-9.815	**<0.001**	
Mean growth ~ Treatment	Intercept	0.0265	0.0034	7.793	**<0.001**	0.033	0.5430	0.1368	3.970	**<0.001**	0.1456
	Treatment	-0.007	0.0045	-1.676	0.0997		-0.5812	0.1835	-3.167	**<0.01**	
Density ovicells ~ Treatment	Intercept	0.0901	0.0267	3.374	**<0.001**	-0.004	0.0751	0.0267	2.816	**<0.01**	0.008
	Treatment	0.0118	0.0400	0.297	0.767		0.0678	0.0396	1.745	0.0827	
Density avicularia ~ Treatment	Intercept	-	-	-	-	-	0.0451	0.0225	1.997	**0.0470**	0.0103
	Treatment	-	-	-	-		0.0630	0.0335	1.880	0.0612	
Damaged zooids ~Treatment	Intercept	0.0491	0.0241	2.039	**0.0426**	0.009	0.0225	0.0205	1.096	0.2742	0.027
	Treatment	0.0630	0.0361	1.745	0.0824		0.0855	0.0305	2.804	**<0.01**	
Damaged zooids ~Treatment* Colony zone	Intercept	0.0645	0.0334	1.928	0.0552	0.031	0.0422	0.0278	1.517	0.1305	0.049
	Treatment	0.111	0.0498	2.247	**0.0256**		0.0103	0.0417	0.249	0.8038	
	Colony zone	-0.0311	0.0477	-0.653	0.5142		-0.0422	0.0408	-1.036	0.3013	
	Treatment* Colony zone	-0.1027	0.0715	-1.436	0.1524		0.1562	0.0604	2.587	**0.0103**	
Area of primary orifice ~ Treatment	Intercept	0.0358	0.0019	18.801	**<0.001**	0.0047	0.0246	0.0012	19.207	**<0.001**	0.0125
	Treatment	0.0041	0.0028	1.433	0.153		0.0038	0.0019	2.022	**0.0443**	
Area of primary orifice ~ Treatment * Colony zone	Intercept	0.0329	0.0026	12.639	**<0.001**	0.056	0.0253	0.0017	14.402	**<0.001**	0.007
	Treatment	0.0003	0.0038	0.080	0.937		0.0039	0.0026	1.482	0.140	
	Colony zone	0.0059	0.0037	1.610	0.1009		-0.0015	0.0026	-0.597	0.551	
	Treatment* Colony zone	0.0080	0.0055	1.445	0.150		-0.00006	0.0038	-0.018	0.983	

Supplementary Table S6. SEM variables (mean ± SE) of colonies between species and treatment in thermal stress experiment and increasing temperature experiment.

Treatment	Sp	Colony zone	Density zooids	Mean area primary orifice	Density damaged zooids	Density ovicells	Mean ovicells size (area)	Density avicularia	Mean avicularia size
Control	*M. truncata*	Proximal part	4±0.54	0.029±0.002	0	0	0	-	-
		Distal part	3.5±0.22	0.044±0.005	0	0.05±0.05	0.063	-	-
T (25°C)		Proximal part	3.56±0.21	0.035±0.005	0	0	0	-	-
		Distal part	3.61±0.26	0.034±0.002	0	0	0	-	-
Control	*P. fascialis*	Proximal part	3.67±0.23	0.021±0.001	0	0	0	0.05±0.05	0.028
		Distal part	3.44±0.11	0.024±0.002	0	0	0	0.11±0.07	0.09±0.07
T (25°C)		Proximal part	1.22±0.36	0.028±0.006	0.33±0.14	0.22±0.22	0.031±0.001	0.44±0.26	0.04±0.004
		Distal part	3.56±0.36	0.031±0.005	0.06±0.02	0.27±0.27	0.033±0.003	0.11±0.11	0.03±0.001
Control	*M. truncata*	Proximal part	3.44±0.21	0.033±0.001	0	0.39±0.16	0.054±0.001	-	-
		Distal part	3.33±0.12	0.039±0.003	0	0.22±0.17	0.038±0.016	-	-
T (30°C)		Proximal part	2.83±0.29	0.033±0.001	0.5±0.21	0.11±0.11	0.07±0.016	-	-
		Distal part	2.61±0.13	0.047±0.004	0.11±0.07	0.44±0.17	0.074±0.009	-	-
Control	*P. fascialis*	Proximal part	3.94±0.056	0.025±0.001	0	0.5±0.32	0.03±0.001	0.33±0.21	0.03±0.003
		Distal part	3.44±0.19	0.024±0.001	0	0.05±0.05	0.023	0	0
T (30°C)		Proximal part	3.16±0.26	0.029±0.004	0	0.17±0.17	0.027±0.001	0.5±0.22	0.04±0.004
		Distal part	3±0.15	0.027±0.001	0.5±0.27	0.72±0.37	0.035±0.004	0.17±0.22	0.03±0.01

Supplementary Table S7. Summary of non-lethal effects on oxygen consumption and mineralogical variables between species in thermal stress experiment (25ºC).

Variable	Treatment	*Myriapora truncata*	*Pentapora fascialis*
		**Mean ± SE**	**Mean ± SE**
**O_2 consumption (µmol/g*day)**	Control	15.24±2.42	14.87±1.59
	T (25°C)	8.77±1.90	10.12±5.01
**wt% $MgCO_3$**	Control	8.16±0.14	9.20±0.17
	T (25°C)	8.39±0.27	9.39±0.18
**wt% Calcite 1**	Control	71.48±0.94	63.69±1.8
	T (25°C)	73.24±1.77	55.35±5.81
**wt% Calcite 2**	Control	27.43±0.21	0
	T (25°C)	26.06±1.94	15.15±24.02
**wt% Aragonite**	Control	1.09±0.38	36.31±1.80
	T (25°C)	0.69±0.35	38.44±3.59

Supplementary Figure S1. Demographic analyses of monitored populations during the mass mortality event.

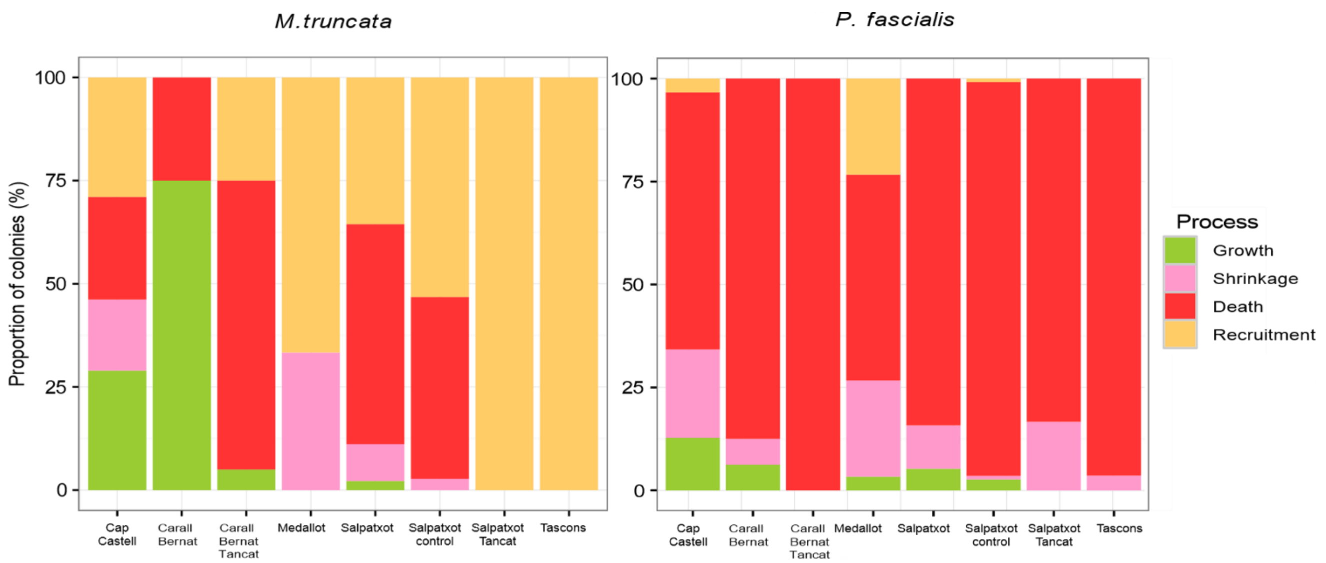

Supplementary Figure S2. Photographic analyses of thermal stress experiment at 25ºC (A) and increasing temperature experiment (B).

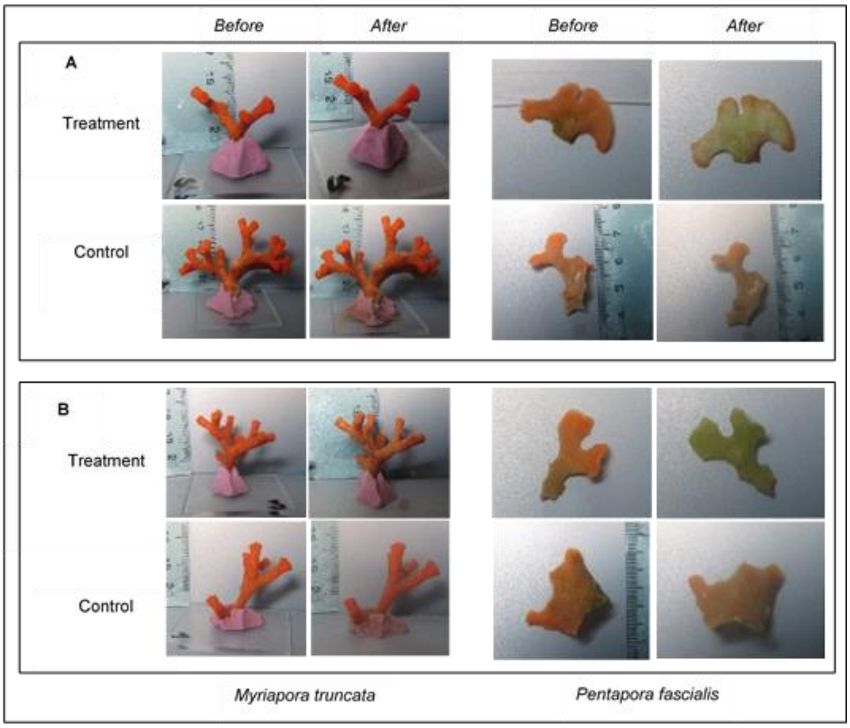

Supplementary Figure S3. Study area and location of monitored localities within the Medes Islands Marine Reserve (points).

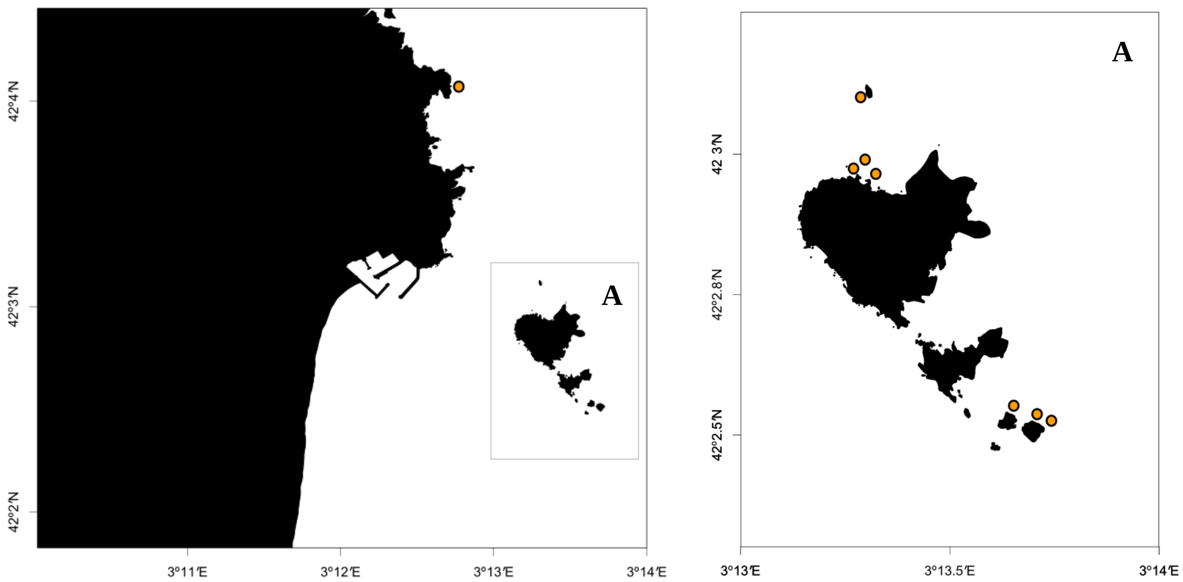

Supplement: Supplementary file 1 — Supplementary Information [file 41598_2018_36094_MOESM1_ESM.pdf]
